# Effect of Amifostine in Head and Neck Cancer Patients Treated with Radiotherapy: A Systematic Review and Meta-Analysis Based on Randomized Controlled Trials

**DOI:** 10.1371/journal.pone.0095968

**Published:** 2014-05-02

**Authors:** Jundong Gu, Siwei Zhu, Xuebing Li, Hua Wu, Yang Li, Feng Hua

**Affiliations:** 1 Tianjin Key Laboratory of Lung Cancer Metastasis and Tumor Microenvironment, Tianjin Lung Cancer Institute, Tianjin Medical University General Hospital, Tianjin, China; 2 Department of Oncology, Tianjin Union Medical Center, Tianjin, China; 3 Department of Human resources, Tianjin Union Medical Center, Tianjin, China; 4 Department of obstetrics and gynecology, Tianjin Hospital of Tianjin City, Tianjin, China; 5 Department of surgery oncology, Shandong cancer hospital, Jinan, China; Ludwig-Maximilians University, Germany

## Abstract

**Background:**

Amifostine is the most clinical used chemical radioprotector, but its effect in patients treated with radiation is not consistent.

**Methods:**

By searching Medline, CENTRAL, EMBASE, ASCO, ESMO, and CNKI databases, the published randomized controlled trials (RCTs) about the efficacy of amifostine in HNSCC patients treated with radiotherapy were collected. The pooled efficacy and side effects of this drug were calculated by RevMan software.

**Results:**

Seventeen trials including a total of 1167 patients (604 and 563 each arm) were analyzed in the meta-analysis. The pooled data showed that the use of amifostine significantly reduce the risk of developing Grade3–4 mucositis (relative risk [RR],0.72; 95% confidence interval [CI],0.54–0.95; *p*<0.00001), Grade 2–4 acute xerostomia (RR,0.70; 95%CI,0.52–0.96; *p* = 0.02), or late xerostomia (RR,0.60; 95%CI,0.49–0.74; *p*<0.00001) and Grade 3–4 dysphagia (RR,0.39; 95%CI,0.17–0.92; *p = *0.03). However, subgroup analysis demonstrated that no statistically significant reduction of Grade3–4 mucositis (RR,0.97; 95% CI,0.74–1.26; *p* = 0.80), Grade 2–4 acute xerostomia (RR,0.35; 95%CI,0.02–5.44; *p* = 0.45), or late xerostomia (RR,0.40; 95%CI,0.13–1.24; *p* = 0.11) and Grade 3–4 dysphagia (RR,0.23; 95%CI,0.01–4.78; *p* = 0.35) was observed in patients treated with concomitant chemoradiotherapy. Compared with placebo or observation, amifostine does not show tumor protective effect in complete response (RR,1.02; 95%CI,0.89–1.17; *p* = 0.76) and partial response (RR,0.90; 95%CI, 0.56–1.44; *p* = 0.66). For the hematologic side effect, no statistical difference of Grade 3–4 leucopenia (RR,0.60; 95%CI,0.35–1.05; *p* = 0.07), anemia (RR,0.80; 95%CI, 0.42–1.53; *p* = 0.50) and thrombocytopenia (RR,0.43; 95%CI,0.16–1.15; *p* = 0.09) were found between amifostine and control groups. The most common amifostine related side effects were nausea, emesis, hypotension and allergic with an average incidence rate (Grade 3–4) of 5%, 6%, 4% and 4% respectively.

**Conclusion:**

This systematic review showed that amifostine significantly reduce the serious mucositis, acute/late xerastomia and dysphagia without protection of the tumor in HNSCC patients treated with radiotherapy. And the toxicities of amifostine were generally acceptable.

## Introduction

Radiotherapy plays a significant role in the management of head and neck squamous cell carcinoma (HNSCC), either for the definitive radiotherapy or for the post-surgical adjuvant radiation[1], [2]. The mucositis, acute or late xerostomia caused by radiation are the most common toxic effects, which usually interrupt the planned course of treatment[3]. Mucositis is an acute non-hematologic toxicity that occurs during the treatment; xerostomia usually develops acutely during chemoradiotherapy and persists for a long period of time. The late side effect always disrupts normal activities such as speaking, eating and may also cause sequelae, including tooth loss and dental caries, which could compromise the quality of patients' life.

For the past several decades, researchers have been investigating use of drugs to decrease the side effects during radiotherapy, so as to increase the amount of radiation that can be safely administered to the patients. The most clinical used radioprotective drug is amifostine that was initially developed as part of the nuclear warfare program[4]. Based on some randomized controlled rials (RCTs), it showed that amifostine could reduce acute and chronic xerostomia in HNSCC patients treated with radiation or concomitant chemoradiotherapy[3], [5]. Hower, some other trials did not demonstrate toxicities reduction effects in course of radiation by adding amifostine to the patients[6], [7]. And another controversy about the use of this drug is the tumor protective effect. Some researchers worried that amifostine may stop tumor tissue responding to radiation and therefore reduce treatment effectiveness[8]. Unfortunately, even after almost 30 years of its development and clinical use, there is still great controversy about its application. Although some reviews state that evidence is not enough to support the tumor protection efficacy of amifostine, the statistical confirmation of those facts has yet to be achieved. In order to answer this question we performed this systematic review and meta-analysis.

## Methods

### Search Strategy

**Figure 1:**
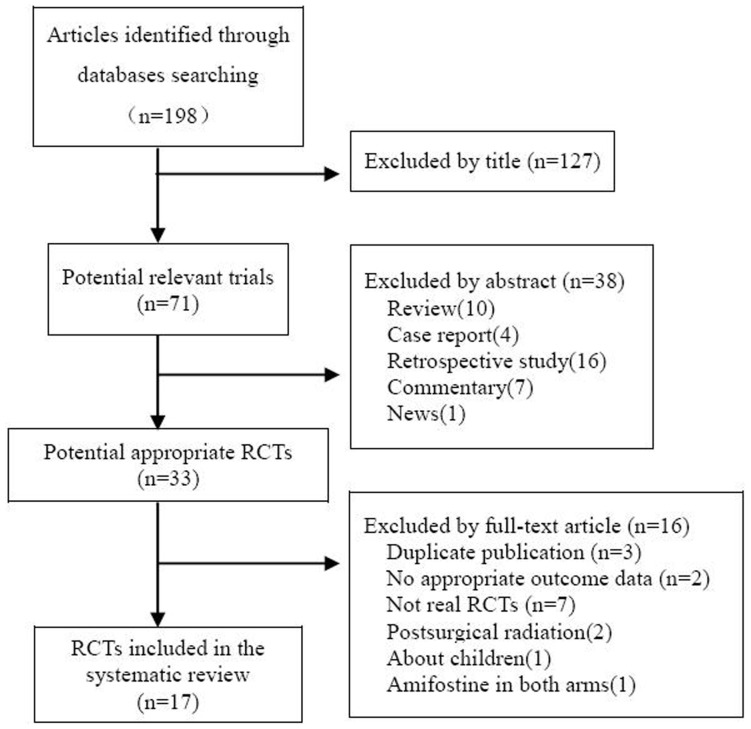
PRISMA Flowchart of the meta-analysis.

The selection procedure of studies was depicted in the PRISMA (Preferred Reporting Items for Systematic Reviews and Meta-Analyses) statement flow chart (Figure 1). Randomized controlled trials comparing radiotherapy vs. radiotherapy plus amifostine for head and neck squamous cell carcinoma, published before January 2012, were identified through an electronic sensitive search of Medline, the Cochrane central register of controlled trials, EMBASE and CNKI databases. The European Society of Medical Oncology (ESMO) and the conference proceedings of the American Society of Clinical Oncology (ASCO) were also searched. The following search terms were used: “Head and neck cancer”, “head and neck carcinoma”, “amifostine”, “Ethyol” “WR-1065”, “WR2721”,“WR1065” or “WR33278”. Searches were limited to human trials, with the language restriction of English and Chinese. All references of relevant articles were scanned for additional analysis.

### Selection Criteria

Details regarding the patients' eligibility criteria, treatment methods and outcomes of the relevant trials were extracted by two reviewer (JG and YW) and then checked by the third reviewer (FH) as described in the Cochrane Handbook for systematic reviews[9]. The patients were limited to locally advanced (Stage III–IV, or inoperable Stage II) head and neck squamous cell carcinoma. The intervention was radiotherapy or chemoradiotherapy plus amifostine and the control was radiotherapy or chemoradiotherapy with placebo or observation. The outcomes were restricted to about the non-hematological toxicity, response rate, hematological toxicity and side effects of amifostine. No surgical excision or neck dissection was performed before the radiation. The RCTs including patients with both HNSCC and other kinds of tumors were also included in the meta-analysis, but data were extracted only for the HNSCC patients.

### Data Extraction and Quality assessment

Data were extracted by two reviewers (JG and HZ) independently from all included RCTs. Any disagreement was consulted to another investigator (YW) for consensus. The general characteristics (name of the first author, year of publication, number of patients, stages, chemoradiation regimens and amifostine dosage), outcomes (non-hematological toxicity: mucositis, acute and late xerostomia, dysphagia; hematological toxicity: leucopenia, anemia and thrombocytopenia; treatment response), and amifostine induced side effects were extracted. The methodological qualities of the trials were assessed by the same investigators (JG and HZ) according to the Cochrane Reviews Handbook 5.0. We pay special attention on the generation and concealment of the sequence of randomization, blinding, incomplete outcome date addresse and selective reports, which generally represent the quality of the RCT[9] in the procedure of trials inclusion.

### Toxicity Assessment

The toxicities caused by radiation were graded according to “Radiation therapy Oncology Group (RTOG) Acute/Late Radiation Morbidity Scoring Criteria” [10]. Acute mucositis and xerostomia were defined as those that occurred up to 90 days after the start of protocol therapy, and late xerostomia was defined as those that occurred 90 days through 12 months after the start of protocol therapy. The treatment response (complete response and partial response) was assessed at about six weeks after the completion of the treatment regimen according to the World Health Organization guidelines.

### Statistical Analysis

Review Manager(RevMan 5.0 provided by The Cochrane Collaboration) was used to do the statistical analysis. Dichotomous data are calculated as the risk ratio (RR) with the 95% confidence interval (CI). The null hypothesis test was considered as no association (RR = 1) between amifostine use and the incidence of radiation toxicity and the tumor response rate. A RR<1 indicates a positive effect of amifostine on the outcome. Statistical heterogeneity of the results across trials was assessed by chi-square (χ^2^) test [11], and the inconsistency was calculated by I^2^ [12]. If heterogeneity was found (χ^2^, *p*<0.05 or I^2^>50%), the random-effect method (Dersimonian-Laird method) was used to pool the data and subgroups analysis was done for further evaluation. Inversely, without significant heterogeneity, fixed-effect method was purchased. The Egger's tests were used for each effect size to evaluate possible publication bias as described by Egger[13].

## Results

### Included Trials and Characteristics

A total of one hundred twelve articles were initially identified by searching the electronic databases. 33 potential applicable studies, published between 1998 and 2010, were retrieved. Of those, sixteen publications were excluded for the reasons: duplicated publication[14]–[16], not real RCT[17]–[23], without appropriate outcome data[24], [25], postsurgical radiation[26]–[28], amifostine in both arms comparing different administration[29], about children[30]. Finally, seventeen trials that included a total of 1167 patients (604 and 563 in each arms) were analyzed in the meta-analysis[3], [5]–[7], [31]–[43]. Eight[6], [31], [32], [34]–[37], [41] of the 17 trials evaluated patients treated with concomitant chemoradiation and the other nine trials [3], [5], [7], [33], [38]–[40], [42], [43] focused on radiation only. Amifostine was delivered intravenously in 13 articles[3], [5]–[7], [31]–[33], [35], [37], [38], [40], [42], [43] and subcutaneously in 4[34], [36], [39], [41]. One trial was a three-arm study that compared different administration schedule of amifostine to observation[7]. The dose of daily amifostine delivered ranged from 200 mg/m^2^ to 400 mg/m^2^. The general characteristics of included trials were outlined in Table 1.

**Table 1:** General characteristics of included randomized controlled trials.

Trials	No.of patiens Ami/Control	Stages included	Daily ami (dose)	Administration	Concomitant Chemotherapy	Radiotherapy
Antonadou[32] (2002)	22/23	III, IV	300 mg/m^2^	IV, 30 minutes before RT, q.d	C (90 mg/m^2^) once per week before RT	60–74Gy, 2 Gy/fraction, 5 fractions weekly
Bourhis[47] (2000)	13/13	IV	300 mg/m^2^	IV, 15–30 minutes before RT, b.i.d	—	62–64 Gy, 2 Gy/fraction, b.i.d with a 8–9 hours interval
Brizel[3] (2000)	143/153	II, III	200 mg/m^2^	IV, 15–30 minutes before RT, q.d	—	50–70Gy, 1.8–2.0 Gy/fraction. q.d
Koukourakis[39] (2000)	19/20	II–IV	500 mg	IH, 20 minutes before RT, q.d	—	44–70Gy,2 Gy/fraction, 5 fractions/week
Fan[38] (2011)	30/26	II–IV	200 mg/m^2^	IV, 15–30 minutes before RT, q.d	—	70–76Gy, 2Gy/fraction, q.d
Peng[37] (2006)	18/19	III, IV	400 mg	IV, 15 minutes before RT, q.d	5-FU(750 mg/m^2^) days:1–3; DDP (50 mg/m^2^) day:5; D (75 mg/m^2^),day:6	74Gy,1.2 Gy/fraction, 2 fractions daily with a 6 hours interval
Yu[42] (2009)	15/15	III, IV	200 mg/m^2^	IV, 20 minutes before RT, q.d	—	70Gy, 2Gy/fraction, q.d
Jiang[41] (2009)	30/30	III, IV	400 mg	IH, 30 minutes before RT.qd	Nedaplatin (100 mg/m^2^) day:1,22 and 43	74Gy, 2Gy/fraction, 5 fractions weekly
He[43] (2004)	17/15	III, IV	200 mg/m^2^	IV, 15–30 minutes before RT, q.d	—	65–74Gy, 2Gy/fraction, q.d
Zhang[40] (2010)	40/40	III, IV	500 mg	IV, 30 minutes before RT, q.d	—	70Gy, 2Gy/fraction, q.d 5 fractions weekly
Braaksma[34] (2005)	27/27	II–IV	500 mg	IH, 15–30 minutes before RT, q.d	P (60 mg/m2) days: 1,8, 15, and 22	72Gy,12Gy/week
Buntzel[35] (1998)	14/14	II–IV	500 mg	IV, 30 minutes before RT. qd	C (70 mg/m2) days:1–5 and days 21–26	60 Gy, 2 Gy/fraction, 5 fractions weekly
Veerasarn[5] (2006)	32/35	II–IV	200 mg/m^2^	IV, 30 minutes before RT, q.d	—	66–70Gy, 2Gy/fraction, q.d
Buentzel[6] (2006)	67/65	II–IV	300 mg/m^2^	IV, 30 minutes before RT, q.d	C(70 mg/m2) Days: 1–5 and 21–25	60–66 Gy, 2 Gy/fraction, 5 fractions weekly
Amrein[31] (2005)	28/8	III, IV	400 mg/m2	IV, 30 minutes before RT, q.d	P (60 mg/m2) days: 1, 8, 15, and 22	70.4 Gy, 1.6 Gy/fraction,2 fractions daily with a 4–6 hour interval
Jellema[7] (2006)	60/31	III, IV	200 mg/m^2^	IV, 30 minutes before RT, 3/5 times weekly	—	63.5–70Gy,2 Gy/fraction, 5 fractions weekly.
Haddad[36] (2009)	29/29	III,IV	500 mg	IH, 30–60 minutes before RT, q.d	C(AUC 1.5) and P (45 mg/m2), weekly for the first 4 weeks of RT	72 Gy, 1.5–1.8Gy/fraction over 6 weeks

Abbreviations:Ami = amifostine; IV =  intravenous injection; IH =  Subcutaneously injection; RT = radiotherapy; C =  carboplatin; 5-FU = 5-fluorouracil; DDP =  Cisplatin; P =  Paclitaxel;

D =  Docetaxel; AUC =  area under the curve; qd = daily; bid = twice daily.

**Figure 2:**
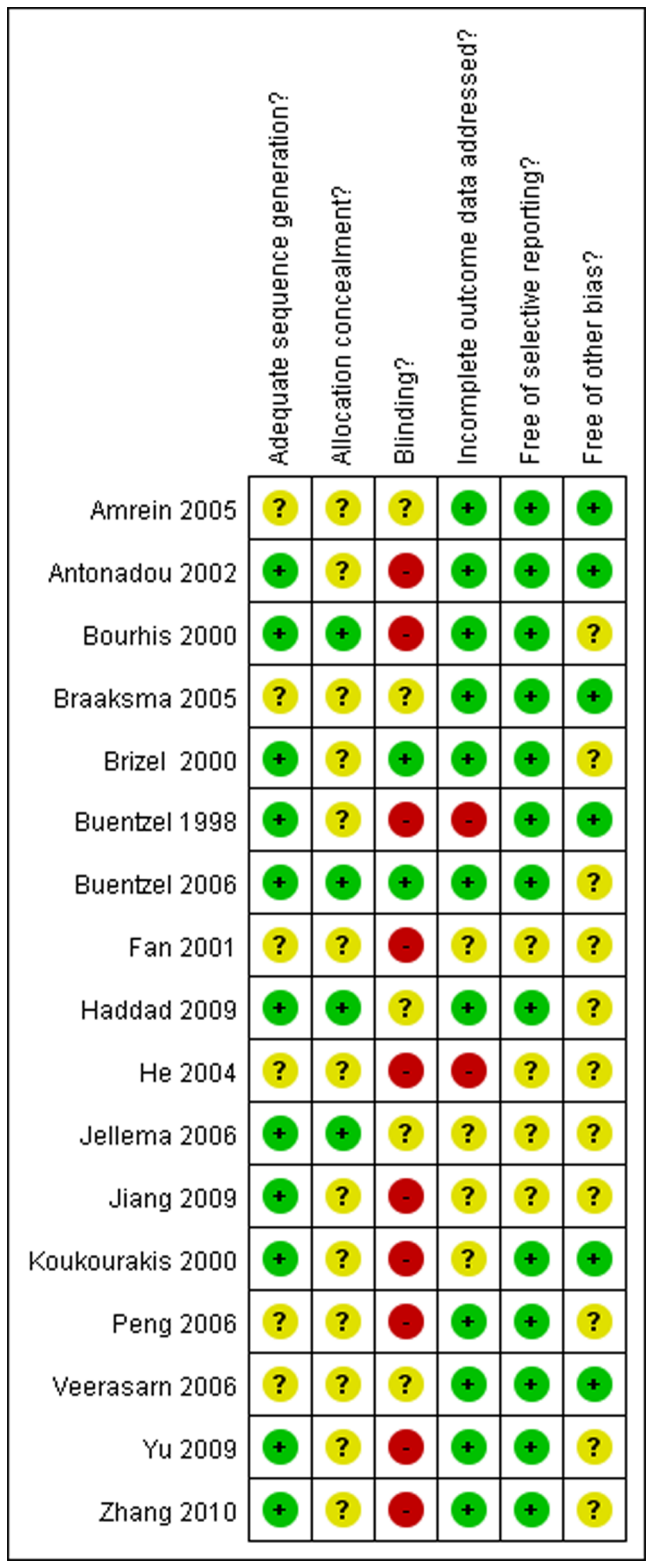
Risk of bias summary. The authors' judgments for each risk of bias item. + is “low risk”; - is “high risk’’;? is “moderate risk.”

The summary of methodological quality was evaluated with a five-question instrument described in the Cochrane Reviews Handbook 5.0. Generally, the qualities of seventeen included trials were considered to be of moderate risk of bias. Randomization was performed in all the 17 trials, but only 4 studies mentioned the allocation concealment[6], [7], [33], [36]. And the adequate sequence generation for randomization was delivered in 11 trials[3], [6], [7], [32], [33], [35], [36], [39]–[42]. For the blinding item, only two trials[3], [6] describe the blindness on evaluators but not on patients and physicians. The outcome of methodological quality for each trial was demonstrated in Figure 2.

### Mucositis

**Figure 3:**
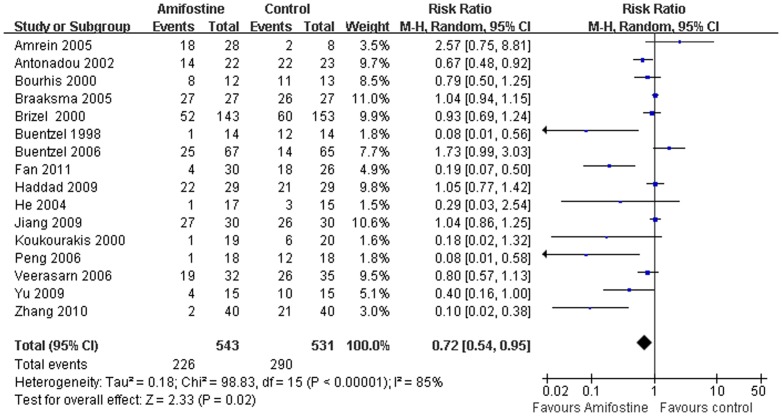
Forest plot of mucositis in HNSCC patients treated with radiotherapy/concomitant chemoradiation. The squares and horizontal lines demonstrate the study-specific OR and 95% CI. The area of the squares reflects the study specific weight (inverse of the variance). The diamond represents the pooled OR and 95% CI.

Of the seventeen trials, 16[3], [5], [6], [31]–[43] evaluated the mucositis with evident heterogeneity between studies(I^2^ = 85%) (Figure 3). The meta-analysis was performed in the random-effect model (Dersimonian-Laird method). Pooled analysis showed that amifostine reduced 28% of Grade 3–4 mucositis (RR,0.72; 95% CI,0.54–0.95; *p*<0.00001) indicating it can significantly decrease the risk of developing serious mucositis in patients treat with radiation. The publication bias was not detected in this subset analysis by Egger's test (*p* = 0.20). Subgroup analysis was performed cording to different treatment regimens (concomitant chemoradiotherapy or radiotherapy only) and admifostine administration (intravenously in or subcutaneously). In the subgroup analysis, statistically significant reduction of mucositis by using of amifostine was observed in patients treat with radiation only (RR,0.49; 95%CI,0.30–0.78; *p* = 0.03) and delivered intravenously(RR,0.52; 95%CI,0.34–0.78; *p* = 0.002) but not in patients treated with concurrent chemoradiation (RR,0.97; 95%CI,0.74–1.26; *p* = 0.80) and administered subcutaneously (RR,1.09; 95%CI,0.94–1.27; *p* = 0.24) (Table 2).

**Table 2:** Subgroup analysis of radiation induced side effects according to treatment strategy.

Subgroups	Mucositis		*p*	Acute xerostomia		*p*	Late xerostomia		*p*	Dysphagia		*p*
	RR	95%CI		RR	95%CI		RR	95%CI		RR	95%CI	
Treatment												
Chemradiation	0.97	0.74–1.26	0.80	0.35	0.02–5.44	0.45	0.40	0.13–1.24	0.11	0.23	0.01–4.78	0.35
Radiation only	0.49	0.30–0.78	0.03	0.69	0.52–0.93	0.02	0.64	0.45–0.91	0.01	0.32	0.17–0.61	0.0004
Administration												
IV	0.52	0.34–0.78	0.002	0,73	0.54–0.97	0.03	0.60	0.49–0.74	0.00001	0.39	0.17–0.92	0.03
IH	1.09	0.94–1.27	0.24	0.08	0–1.34	0.08						

### Acute xerostomia

**Figure 4:**
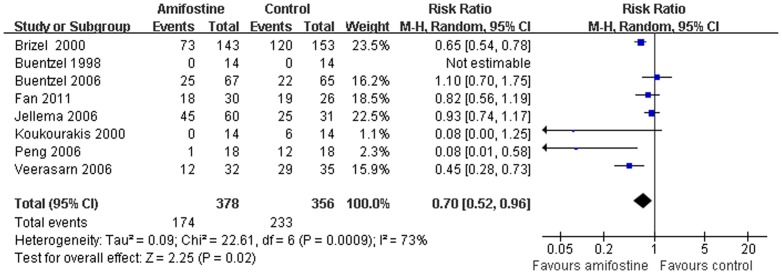
Forest plot of acute xerostomia in HNSCC patients treated with radiotherapy/concomitant chemoradiation. The squares and horizontal lines demonstrate the study-specific OR and 95% CI. The area of the squares reflects the study specific weight (inverse of the variance). The diamond represents the pooled OR and 95% CI.

Eight trials[3], [5]–[7], [35], [37]–[39] report the number of patients developed acute xerostomia in both arms. The heterogeneity between the trials was obvious. Pooled analysis with the random-effect model demonstrated that significant reduction of Grade 2–4 acute xerostomia was achieved suing amifostine in patients with HNSCC (RR,0.71; 95%CI,0.52–0.96; *p* = 0.02) (Figure 4). No publication bias was found in the acute xerostomia analysis (Egger's test, *p* = 0.76). But in the subgroup analysis, use of amifostine does not reduce the risk of developing acute xerostomia in patients receiving concomitant radiation (RR,0.35; 95%CI,0.02–5.44; *p* = 0.45) (Table 2).

### Late xerostomia

**Figure 5:**
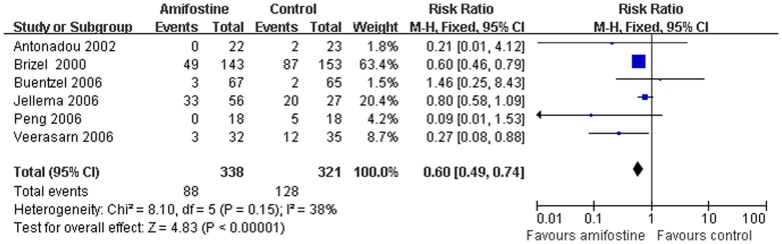
Forest plot of late xerostomia in HNSCC patients treated with radiotherapy/concomitant chemoradiation. The squares and horizontal lines demonstrate the study-specific OR and 95% CI. The area of the squares reflects the study specific weight (inverse of the variance). The diamond represents the pooled OR and 95% CI.

Late xerostomia were reported in six trials[3], [5]–[7], [32], [37] without the heterogeneity between the studies (I^2^ = 38%). Meta-analysis showed amifostine significant reduced 40% risk (RR,0.60; 95%CI,0.49–0.74; *p*<0.00001) of Grade 2–4 late xerostomia compared with placebo or observation (Figure 5). Subgroup analysis also showed that amifostine does not reduce the risk of developing late xerostomia in HNSCC patients treated with radiotherapy plus chemotherapy (*p* = 0.11) (Table2).

### Dysphagia

Data of dysphagia was pooled from five [5], [32]–[35] of the 17 included trials. Compared with the control, use of amifostine significant decrease the risk of developing Grade 3–4 dysphagia (RR,0.39; 95%CI,0.17–0.92; *p* = 0.03). Extreme heterogeneity between the studies was found (I^2^ = 94%). However, all the trials included in the analysis of dysphagia had an estimate point that favored amifostine, and three of them reached statistical significance. We preferred that amifostine significant decrease the risk of developing Grade 3–4 dysphagia.

### Treatment response

**Figure 6:**
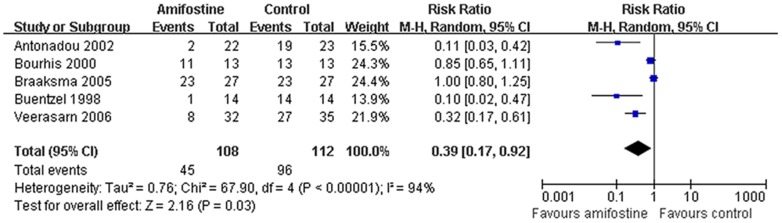
Forest plot of dysphagia in HNSCC patients treated with radiotherapy/concomitant chemoradiation. The squares and horizontal lines demonstrate the study-specific OR and 95% CI. The area of the squares reflects the study specific weight (inverse of the variance). The diamond represents the pooled OR and 95% CI.

Response rates were available from six trials[31], [32], [34], [35], [37], [42]. No statistical heterogeneity between studies was found by Egger's test in both complete (I^2^ = 21%) and partial (I^2^ = 0%) response analysis. The pooled RR estimate for the complete and partial response was 1.02 (95%CI, 0.89–1.17; *p* = 0.76) and 0.90 (95%CI,0.56–1.44; *p* = 0.66) respectively (Figure 6). Neither of the them reached statistical significance (Figure 6).

### Hematological toxicity

**Figure 7:**
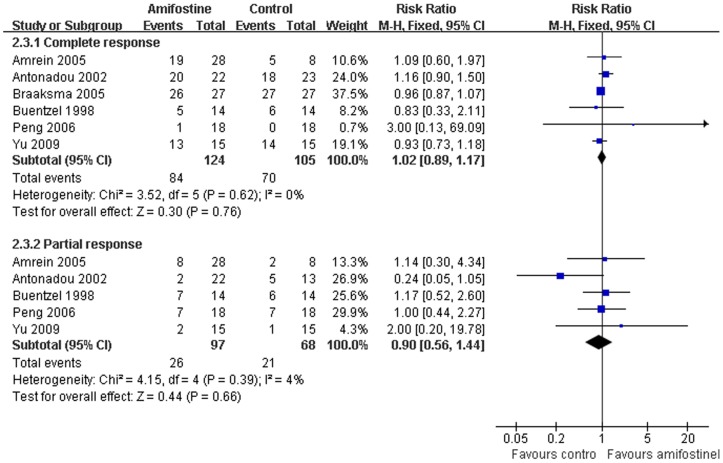
Forest plot of acute response rate in HNSCC patients treated with radiotherapy/concomitant chemoradiation. The squares and horizontal lines demonstrate the study-specific OR and 95% CI. The area of the squares reflects the study specific weight (inverse of the variance). The diamond represents the pooled OR and 95% CI.

Hematological toxicity, including leucopenia, anemia and thrombocytopenia were extracted in five trials[5], [6], [32], [35], [37] without heterogeneity between the studies (I^2^ = 38%). Meta-analysis showed that no statistical difference of Grade 3–4 leucopenia (RR, 0.60; 95%CI,0.35–1.05; *p* = 0.07), anemia (RR,0.80; 95%CI,0.42–1.53; *p* = 0.50) and thrombocytopenia (RR, 0.43; 95%CI, 0.16–1.15; *p* = 0.09) were found between amifostine and control groups. Publication bias was not observed by Egger's test (*p* = 0.63) (Figure 7).

### Side effects of Amifostine

**Figure 8:**
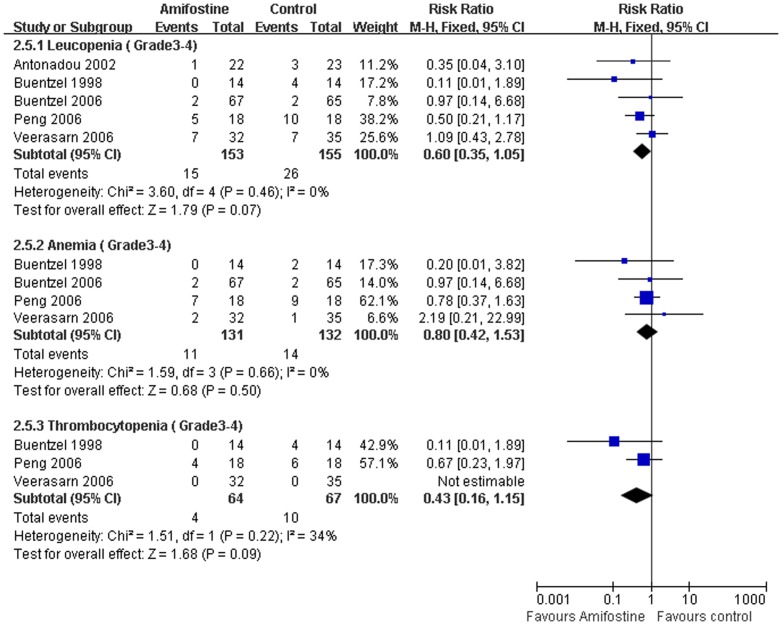
Forest plot of hematological toxicity in HNSCC patients treated with radiotherapy/concomitant chemoradiation. The squares and horizontal lines demonstrate the study-specific OR and 95% CI. The area of the squares reflects the study specific weight (inverse of the variance). The diamond represents the pooled OR and 95% CI.

Eight trials[3], [5]–[7], [32]–[35] describe the amifostine toxicity. The most common amifostine related side effects were nausea, emesis, transient hypotension and allergic. The average incidence rate of them (Grade 3–4) was 5%, 6%, 4% and 4% respectively (Figure 8). Of the eight trials reported the amifostine-induced toxic effects, seven studies[3], [5]–[7], [32], [33], [35] delivered the this drug by intravenous injection and only one [34] by subcutaneous injection, indicating that hypodermic administration of the drug may substantially reduce these side-effects[44].

## Discussion

About half a million cases of head and neck squamous cell carcinomas are diagnosed yearly around the world[45]. The majority of the new cases of head and neck cancer have locally advanced disease. Management of these patients is complex because the vital structures in this area. Radiotherapy or chemoradiation is the most common used strategy at this stage. However, the radiation-related toxicities are inevitable and sometimes vital with the increase of radiotherapy dose. In the past several decades, researchers have been investigating use of drugs to decrease the side effects during radiotherapy, in order to increase the amount of radiation that can be safely administered to the patients. Amifostine is one of the radioprotector initially developed as part of the nuclear warfare program. Some randomized controlled trials demonstrated that this drug reduced some of severity radiation-related toxic effects (acute or late xerostomia) without tumor protection. However, some other trials do not support this. So, we performed this systematic review and meta-analysis to compile the inconsistent evidence together to evaluate the true clinical efficacy of this drug.

In this meta-analysis, we generally find that use of amifostine significantly reduced the radiation-induced toxicities of serious mucositis(*p*<0.00001), acute or late xerostomia(*p* = 0.02), and dysphagia(*p*<0.00001). However, the subgroup analysis, according to the treatment regimen (radiation only or concurrent chemoradiotherapy), demonstrate that patients receiving concomitant chemoradiation can not benefit reduction of side effects from use of amifostine. Heterogeneity between trials in this subgroup analysis set was found. And the trials included in this subset had inconsistent estimate points that statistically significant favored the amifostine or placebo/observation. So, the definitive efficacy of this drug in HNSCC patients receiving concomitant chemoradiation is conservative.

Two major controversy for clinical use of amifostine is tumor protection and its toxicity. Some oncologists argued that amifostine may reduce treatment effectiveness by stopping tumor tissue responding to radiation[8]. Morever, some opponents point out that there are not enough evidence to justify the conservative tumor protective effects of this drug[46]. However, considering the realities of randomized controlled trials and practice, absolute rejection the tumor protective effects compromised by amifostine is difficult. To demonstrate a hypothetical 40%–45% reduction of survival odds (α = 0.05; 80% power), 2492 patients would require in the trial, which was highly impractical[46].Under this situation, meta-analysis was conducted where possible to gain an objective consensus from repeatedly inconsistent trials. In this systematic review, we did not found statistical significant difference in complete response (RR,1.02; 95%CI,0.89–1.17; *p* = 0.76) and partial response (RR,0.90; 95%CI,0.56–1.44; *p* = 0.66) between the groups. However, without enough individual data, the disease-free survival (DFS) and overall survival (OS) were not pooled in this meta-analysis. However, Bourish *et al* [47] published a meta-analysis about effect of amifostine on survival among patients treated by radiotherapy with individual patients data recently. In that meta-analysis, twelve randomized trials consisted of 1119 patients were analyzed in the study and the result showed that amifostine did not reduce OS and DFS in patients treated with radiotherapy or concomitant chemoradiotherapy. So, we concluded that amifostine does not protect the tumor during radiation.

Another controversial issue about amifostine is toxicities induced by itself. The most common side effects of this drug were nausea, vomiting or transient hypotension, ranging from 5% to 30% according to the literatures[7], [32], [33]. Our study showed that the average incidence of Grade 3–4 nausea, emesis, hypotension and allergic were 5%, 6%, 4% and 4% respectively. Of the nine trials that reported the amifostine-induced toxic effects, 8 delivered the drug by intravenous injection and only one by subcutaneously, indicating that subcutaneous administration of this drug may substantially reduce these side-effects. However, we also find, in the subgroup analysis, that significant reduction of Grade 3–4 mucositis can not be reached in patients receive amifostine subcutaneously(*p* = 0.24).

The results of this meta-analysis were based on published randomized controlled trials not on individual patients' data. The results should therefore be interpreted cautiously. Although no evident of publication bias was found in this study by Egger's test, the small number of trials and possible existence of unpublished studies are inevitable and completely ruling out this possibility in all aspects is difficult[48].

In conclusion, this systematic review demonstrates that amifostine concurrently with RT significantly reduced the side effects of serious mucositis, acute/late xerastomia and dysphagia without protection of the tumor in HNSCC patients. The reduction of radiation-induced toxicities by amifostine should be weighed against the toxicities of amifostine itself according to the individual treatment strategy. Therefore, amifostine does have a role during radiation, a role that is still evolving. Well-designed RCTs are the necessary investments that will further explore the potential benefits of amifostine.

## Supporting Information

Checklist S1 PRISMA Checklist.
(DOC)
